# Assessment of Iodine Status in Iranian Students Aged 8–10 Years: Monitoring the National Program for the Prevention and Control of Iodine Deficiency Disorders in 2016

**Published:** 2020-02

**Authors:** Mansoureh REZAIE, Sepideh DOLATI, Alemeh HARIRI FAR, Zahra ABDOLLAHI, Said SADEGHIAN

**Affiliations:** 1. Department of Nutrition, Ministry of Health, Tehran, Iran; 2. Department of Community Nutrition, Faculty of Nutrition, Tabriz University of Medical Sciences, Tabriz, Iran; 3. Research Institute for Endocrine Sciences, Shahid Beheshti University of Medical, Tehran, Iran

**Keywords:** Iodine deficiency, Iodized salt, Student, Iran

## Abstract

**Background:**

Iodine is a key element in the synthesis of thyroid hormones. The deficiency of the secretion of them will Reduce IQ, disturbance in the psychomotor growth and shortened height. Urinary iodine is a good indicator of iodine intake status. Urinary iodine status in at-risk groups is one of the most important indicators of community status.

**Methods:**

All 56 universities/medical faculties in Iran should determine and report median urinary iodine and its relative distribution in school children aged 8 to 10 yr, to determine the status of urine output. The sample size in each university/college is 240 students and the cluster sampling method (48 clusters in each area in 2016) and based on probability Measurement. The amount of urinary iodine was measured quantitatively by acid digestion.

**Results:**

The mean urinary iodine excretion was estimated at 18.61 μg/dl. The median urinary iodine concentration in 52 universities was sufficient, and the national mean of urinary iodine excretion rate was 19.3 μg/dl. The iodine status was estimated in the optimal range in 65.6% of the students and in only 4.7% in the range of moderate and severe deficits, based on the urinary iodine index.

**Conclusion:**

Iodine is sufficient in most parts of the country. The implementation of the country’s national program for the prevention and control of iodine deficiency disorders has made more important the quality control of the collaborative laboratories of this program than before. Moreover, it is absolutely essential to avoid excessive iodine in order to prevent possible side effects.

## Introduction

Iodine is a key element in the synthesis of thyroid hormones. According to the physiological effects of these hormones on the performance of all body cells, effects and consequences of iodine deficiency can causes a heavy burden on the community (1) such as disruption in embryonic development until puberty, results in decreased intelligence, disturbance in psychomotor growth and shortened height (2) that due to significant advances in this field are preventable (3–5).

At present, salt iodization program is covered about 75% of the world's population (6) and countries with iodine deficiency declining from 54 countries in 2003 to 32 countries in 2011 (7, 8).

However, iodine deficiency and it disorders are a major health problems in the world, and the WHO reported a global prevalence (in 126 countries) of goiter was 15.8%, (from 4.7% in the United Provinces to 28.3% in Africa) in 2005, (9) and 31.5 % of students (266 million) and about 2 billion people in the world suffering from low iodine intake (4) in 2007.

Iran has a long history of iodine deficiency and it is a successful country in prevention and controls the iodine deficiency disorders (IDD). The first study on the prevalence of goiter in Iran was performed in 1968, which reported a high prevalence of goiter (10). Further studies done in the 1980s, revealed iodine deficiency and its disorders as a major health problem in Iran (7, 11–14).

Then, the Iranian Ministry of Health began the Salt iodization program (10–40 ppm) and the generalization of iodine salt intake, with the establishment of the Iranian National Committee for Control of IDD in 1989. At the same time, it integrates activities, such as monitoring the level of iodine at the level of production and supply of salt, monitoring the level of iodized salt consumption at household level, monitoring the annual urinary iodine in students aged 8 to 10 yr and training of health workers and public to sustainable remove of IDD (7). According to the results of three national studies conducted in 1996, 2002 and 2007, the median urinary iodine excretion rate was reported above 10 μg/dL in all three studies, and the prevalence of goiter was less than 10%, since 2002 (15–17).

It is essential to continuation of programs such as monitoring the status of iodized salt, household consumption and control of the status of receiving high-risk groups to eradicate iodine deficiency and successful prevention and control (7).

Urinary Iodine is a good indicator to determine the status of iodine since more than 90% of iodine intake is excreted through the urine (18). Urinary Iodine is one of the most important indicators of community status, in at-risk groups such as children. For this reason and based on WHO/UNICEF/ICCIDD (Iranian National Committee for Control of IDD) recommendations, the median urinary iodine in 8 to 10 yr old students are measured at fixed intervals to make sure that the amount of iodine intake is sufficient (100) and its annual survey is based on the National Program for Prevention and Control of Disorders due to iodine deficiency in Iran.

The most important indicators of prevention and control of IDD are the median urinary iodine concentration above 10 μg/dL and the control of the prevalence of moderate deficiency (based on the percentage of the population with iodine deficiency of 5 μg/dL) in less than 20% of all provinces. This survey should be done annually in the population covered by the medical sciences universities of Iran (7).

This study presented the moderate urinary iodine status in students aged 8 to 10 yr in Iran in the school year 2016–2017.

## Materials and Methods

This study is obtained from the universities reports to Community Nutrition Improvement Office in the Ministry of Health in Iran, during the school 2016–2017. These reports are including the median urinary iodine and the relative distribution of urinary iodine in students 8 to 10-year-old in Iran.

According to the Iranian National Committee for Control of IDD (INCCI), all universities/medical faculties (56 universities or colleges) are required to determine the iodine intake and its relative distribution in the population of 8 to 10 yr old students, covered by the following five categories based on WHO recommendations: Less than 2 μg/dl (severe iodine deficiency), 2.0 to 4.9 μg/dL (moderate iodine deficiency), 5.0 to 9.9 (mild iodine deficiency), 29.9 to 10 μg/dL (favorable) and equal or greater than to 30 micrograms per deciliter (excessive intake).

The sample size was 240 students in each university/college (based on the decision INCCI) and the cluster sampling method (48 clusters in each area) used in this study based on the probability based on the measurement.

In each region, 48 schools - based on the last list of primary schools - were selected. Based on urban and rural population and equal to gender, and, five students aged 8 to 10 were randomly assigned to this study in each school.

The sampling was done using plastic containers and urinary iodine concentration was measured quantitatively by acid digestion (19). The measurement method was the same in all provinces.

There was a laboratory in each university/college and the technical staff of the laboratory was trained at the beginning of the year. The Department of Endocrinology and Metabolism of the Shahid Beheshti University was responsible for training and supervising at universities.

After sampling (autumn 95 to spring 96), all universities analyzed their data and were sent the results to the Nutrition Department of Ministry of Health, Iran. These results included median urinary iodine and distribution of urinary iodine in 5 ranges; less than 2 micrograms per deciliter, 2–4.9 μg/dl, 5 – 9.9, 10 – 29.9 μg/dl, and equal or more than to 30 μg/dl.

There are 31 provinces in Iran. In 20 provinces, the total populations are under the supervision of a university. While in 11 other provinces, there are two or more universities in each province, and the population of each province is distributed among several universities. In the first case (20 provinces), the reports of each university indicate the status of the province's total iodine population. While in 11 other provinces, to determine the relative distribution of urinary iodine, the ratio of the population of each university to the total population of the province has been calculated.

After determination the relative distribution of iodine (based on the amount of urine iodine) by universities, the iodine status was estimated throughout the country according to percentage of the population of each province to the population of the country.

## Results

Table 1 shows the median urinary iodine in 56 universities of medical sciences. At least 240 students aged 8 to 10 yr have been screened for urine samples in each university, and in the country, 13,428 urine sample have been examined for urinary iodine. The mean urinary iodine excretion in all samples was estimated at 18.61 μg/l.

**Table 1:** The median urinary iodine (μg/dl) in 56 countries of the country's medical sciences

***Province***	***University***	***Numbers***	***Mean***	***Median***	***Iodine nutritional status***
East Azerbaijan	Tabriz	240	14	13	Adequate
	Maragheh	240	17	14	Adequate
West Azerbaijan	Orumieh	240	21	15	Adequate
Ardebil	Ardebil	235	19	19	Adequate
Isfahan	Isfahan	240	15	13	Adequate
	Kashan	240	16	14	Adequate
Alborz	Alborz	240	24	26	Adequate
Ilam	Ilam	235	22	19	Adequate
Boushehr	Bushehr	243	20	20	Adequate
Tehran	Tehran	240	21	20	Adequate
	Shahid Beheshti	240	20	18	Adequate
	Iran	240	17	15	Adequate
Chahar Mahal Bakhtiari	Shahrekord	243	20	19	Adequate
North Khorasan	Bojnourd	240	8	7	Inadequate
	Esfarayen	240	9	9	Inadequate
Khorasan Razavi	Mashhad	241	15	14	Adequate
	Neishabour	240	15	13	Adequate
	Sabzevar	239	19	19	Adequate
	Gonabad	240	11	9	Inadequate
	Torbat Heidarieh	246	12	7	Inadequate
	Torbat Jam	217	12	10	Adequate
Southern Khorasan	Birjand	240	12	10	Adequate
Khuzestan	Ahvaz	240	27	26	Adequate
	Abadan	240	13	13	Adequate
	Behbahan	240	24	27	Adequate
	Dezfoul	240	16	17	Adequate
Zanjan	Zanjan	253	20	20	Adequate
Semnan	Semnan	240	15	14	Adequate
	Shahroud	240	22	20	Adequate
Sistan and Baluchestan	Zahedan	283	17	16	Adequate
	Zabol	240	15	13	Adequate
	Iranshahr	240	17	15	Adequate
Fars	Shiraz	239	21	20	Adequate
	Jahrom	240	16	15	Adequate
	Fasa	240	16	15	Adequate
	Larestan	216	20	19	Adequate
	Gerash	246	23	22	Adequate
Qazvin	Ghazvin	240	14	13	Adequate
Qom	Gom	221	13	14	Adequate
Kordestan	Kordestan	269	26	25	Adequate
Kermanshah	Kermanshah	240	15	14	Adequate
Kohgiloyeh and Boyerahmad	Yasuj	212	18	18	Adequate
Kerman	Kerman	240	22	21	Adequate
	Rafsanjan	235	20	19	Adequate
	Jiroft	212	26	25	Adequate
	Bam	240	19	17	Adequate
Golestan	Golestan	229	24	18	Adequate
Gilan	Gilan	240	22	20	Adequate
Lorestan	Lorestan	232	29	30	Adequate
Markazi	Markazi	240	24	23	Adequate
	Saveh	241	17	15	Adequate
Mazandaran	Mazandaran	250	21	20	Adequate
	Babol	240	31	34	Adequate
Hormozgan	Hormozgan	261	23	21	Adequate
Hamedan	Hamedan	246	19	18	Adequate
Yazd	Yazd	240	20	16	Adequate

Moreover, median urinary iodine excretion was insufficient range in 52 universities (urinary iodine excretion equal to or greater than 10 μg/dl), but in 4 universities (all located in the northeast of Iran), was in the range of mild deficiency.

Urinary iodine excretion was in the optimal level (between 10–29 μg/dl) in 65.4% of the samples and it is below 2 μg/dl, in less than 1% of the population. Urinary iodine excretions were reported in 4.1% and 15 % of samples, 2–9.9 μg/dl and 5–9.9 μg/dl, respectively.

**Fig. 1:**
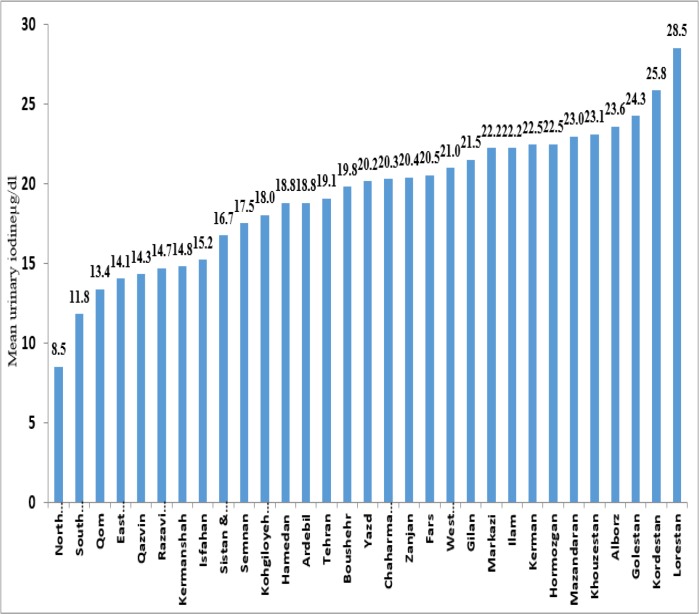
Mean urinary iodine excretion (μg/dl) in 10-8 year old students in different provinces of Iran Annual National Monitoring Report on Urinary Iodine Students in the 2013–14 academic years

Subsequently, the university information was weighted according to the population of children aged 8 to 10 from each university to the total number of children in this age group in the same province. The mean urinary iodine excretion in 30 provinces of 31, is in the range of 10 to 30 μg/dl, the lowest mean urinary iodine excretion was in Southern Khorasan province and the highest was Lorestan (Fig. 1). The mean of urinary iodine excretion rate estimates 19.3 μg/dl in Iran.

**Fig. 2:**
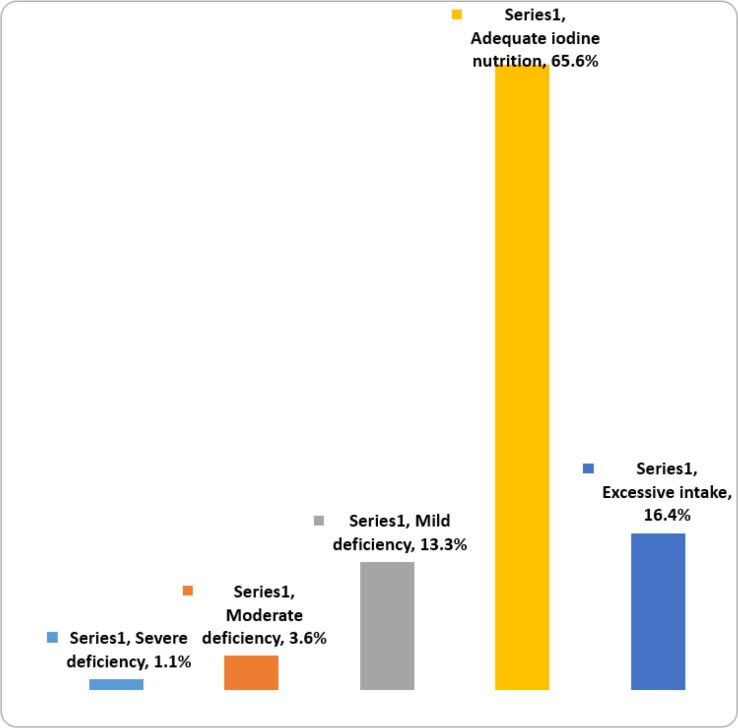
Distribution of iodine deficiency based on the amount of urinary iodine in 8–10 year old students in the country (Annual National Monitoring Report on Urinary Iodine Students in the 1984–2014 academic years)

Based on urinary iodine index, the findings showed iodine status was in optimal range in 65.6 % students and 4.7 % in moderate and severe deficits (Fig.2).

**Fig. 3:**
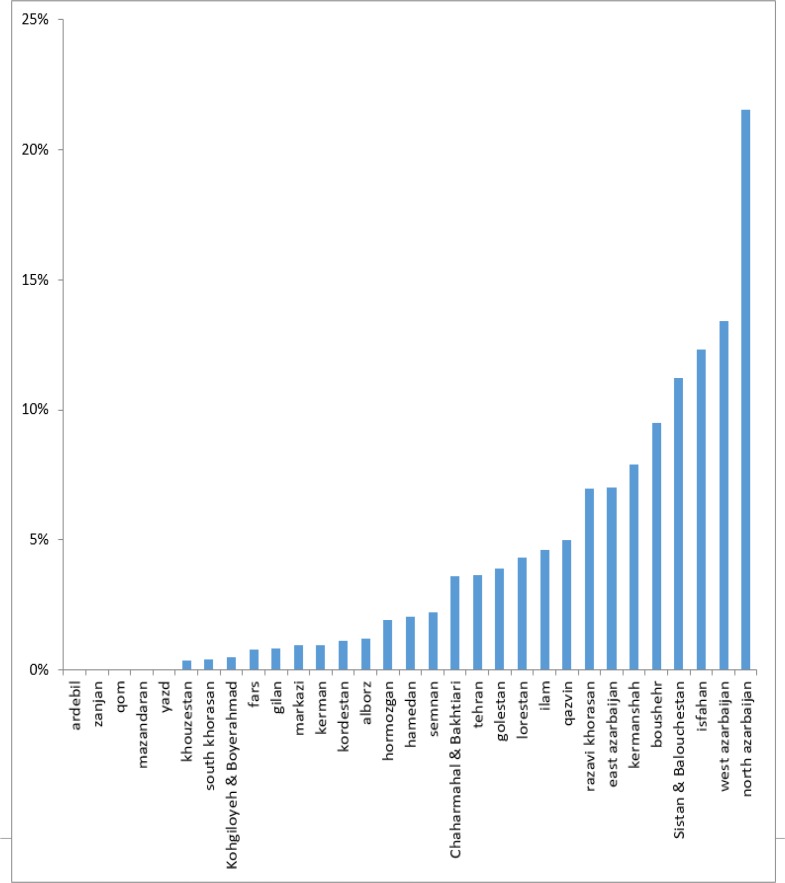
The prevalence of mild and moderate iodine deficiency in the 8–10 yr-old students’ country (Annual National Monitoring Report on Urinary Iodine Students in the 1984–2014 academic years)

The highest prevalence of mild to moderate deficiency was observed in North Khorasan (21.5 %), Uremia (13.4%) and Isfahan (12.3%). The prevalence of mild to moderate iodine deficiency was less than 1% in 12 provinces (Fig. 3).

**Fig. 4:**
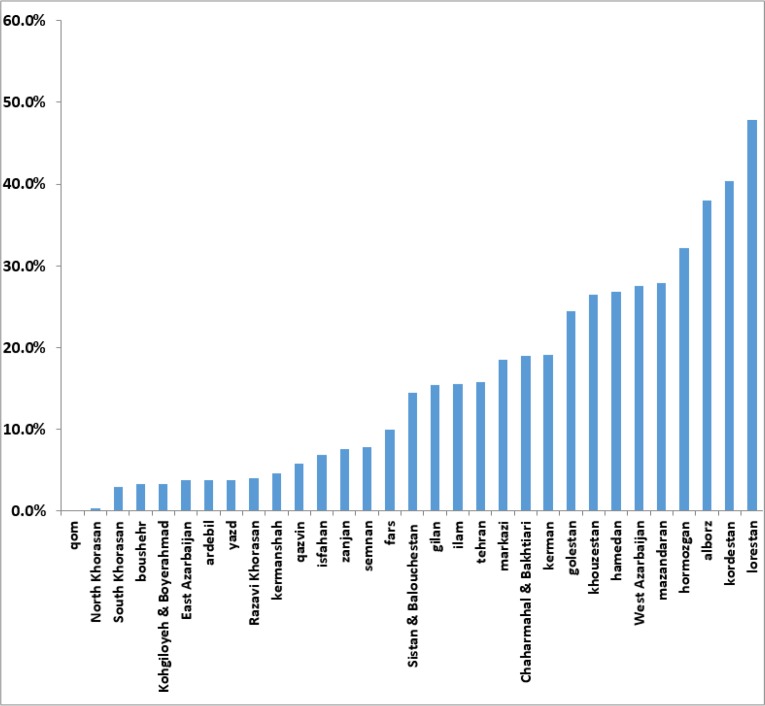
Distribution of excess iodine intake in 8–10-year-old students in the country (Annual National Monitoring Report on Urinary Iodine Students in the 1984–2014 academic years)

Determination of urinary iodine showed that 16.4 % of students get excess iodine intake (equal to or greater than 30 μg/dl) (Fig. 2). The highest percentage of samples with urinary iodine excretion over 30 μg/dl of the provinces of Lorestan and Kurdistan (Fig. 4).

## Discussion

The annual monitoring of urinary iodine is a tool for monitoring the national program for the prevention and control of iodine deficiency disorders in the academic year of 1994–95. The results of this report showed Iodine intake is sufficient at the national level and in the majority of provinces.

According to the reports submitted by the universities, it was not possible to calculate the median urinary iodine level at national level. As a result, we calculated the mean of urinary iodine excretion. The national mean of urinary iodine excretion was 19.3±5.8 μg/dl. This amount was higher than the median urinary iodine in the study of 1393 (20), which the mean urinary iodine was 16.1 μg/dl for the country.

In the present study, iodine deficiency was estimated at only 4.7% in school children aged 8 to 10, This amount was lower than the reported results in the fifth national survey (20). In that study,10% of the students had urinary iodine excretion lower than 10 μg/dl.

The results of our study indicated that the median urinary iodine was desirable (10μg/dl or more) in 30 provinces and the population covered by 52 universities in the country. But at the same time, it is low in North Khorasan province and Torbat Heydarieh, Gonabad, Esfarayen and Bojnourd universities, which is the debate.

In a study (20), the median urinary iodine was not less than 10 μg/dl in any of the provinces of Iran, and it was 13.7 μg/dl in the northern Khorasan Province. The prevalence of iodine deficiency was showed 21.5% in North Khorasan, in our study, which is different from the previous one. The reason for this difference is probably the measurement of urinary iodine or Real iodine deficiency.

The ICCIDD has to develop the program of quality control for the collaborative laboratories of the national program. Moreover, equipping laboratories and training and retraining experts in partner labs requires review and development. The laboratory kits and the accuracy of laboratory performance should be checked. Moreover, it is necessary to reduce the number of peer labs to make it easier to manage for the committee. The reported median urinary iodine in Lorestan (30 μg/dl) and Babol (34 μg/dl) universities are another issues that needs to be addressed. In the fifth national monitoring program, the median urinary of iodine was reported 14.6 μg/dl and 12.5 μg /dl, in Lorestan and Mazandaran provinces (covered by the Babol University of Medical Sciences), respectively. Based on this finding, the result of the current study is very different from the previous study.

In general, although, the results show the adequacy of iodine intake at national level, and there are in line with the results of national studies in this regard (15–17), but the following points should be made the attention for policymakers in annual urinary iodine monitoring program in school children.

Although it is possible that the results reported by the Lorestan and Babol universities are due to laboratory error in measuring iodine, however, there are concerns over the side effects of excessive iodine intakes after two decades of salt iodization. Based on the fifth National Surveillance Survey (20), the culture of iodized salt consumption has improved significantly in the country. As, currently, 98% of Iranian households use iodized salt. The national studies in Iran (21) have shown sodium intake is higher than standard values, in Iranian children aged 3 to 10, which raises concerns about excessive iodine intake in this population

The National Committee for the Prevention and Control of Iodine Deficit Disorders has informed the controlling policies such as not using iodized salt in cooking bread and the lack of iodized salt in the food industry, To control the side effects of excessive iodine intake, along with the design and implementation of iodine salt enrichment program. These policies should be supervised in future by National Committee on prevention and control of iodine deficiency disorders.

## Conclusion

Iodine intake at national level are favorable and consistent with the results of national studies. Despite the probability of laboratory error from the universities of Lorestan and Babol, it seems that the current main issue in this field is overdose of iodine intake.

## Ethical considerations

Ethical issues (Including plagiarism, informed consent, misconduct, data fabrication and/or falsification, double publication and/or submission, redundancy, etc.) have been completely observed by the authors.
